# Retraction: The mediating roles of personal mastery and health-promoting behaviors in the relationship between self-regulatory fatigue and quality of life among patients with type 2 diabetes mellitus

**DOI:** 10.1371/journal.pone.0362331

**Published:** 2026-11-09

**Authors:** The PLOS One Editors

Following publication of [1], the corresponding author ZW contacted PLOS requesting retraction of their article due to errors in the methodology and difficulty reproducing the data.

During editorial follow-up, it was also determined that this article contains a substantial degree of overlap with a previously published article [2] with regard to the reported abstract, Figs 1-2, and Tables 1-6. An editorial assessment by PLOS has established that the extent of the overlap is such that this article [1] constitutes redundant publication.

The *PLOS One* Editors retract this article [1] because, per our editorial assessment, it does not meet *PLOS One’s* publication criterium (#2) [3], and because of the above concerns that remain unresolved and question this study’s compliance with the PLOS Ethical Publishing Practice policy [4].

In light of the duplicate publication concerns, PLOS did not investigate the merit of the data reproducibility and methodology concerns further.

All authors agreed with the retraction.
